# Correction: Evaluating the impact of multivariate imputation by MICE in feature selection

**DOI:** 10.1371/journal.pone.0261739

**Published:** 2021-12-16

**Authors:** Maritza Mera-Gaona, Ursula Neumann, Rubiel Vargas-Canas, Diego M. López

The institution that the second author, Ursula Neumann, is affiliated with has updated their name. The correct affiliation is: Fraunhofer IIS, Fraunhofer Institute for Integrated Circuits IIS, Division Supply Chain Services, Group Data Science.

## References

[1] Mera-Gaona M, Neumann U, Vargas-Canas R, López DM (2021) Evaluating the impact of multivariate imputation by MICE in feature selection. PLoS ONE 16(7): e0254720. doi: 10.1371/journal.pone.0254720 34320016 PMC8318311

